# Supplementary material for: Identification of a stereotypic molecular arrangement of endogenous glycine receptors at spinal cord synapses
Source: eLife. 2021 Dec 8;10:e74441. doi: 10.7554/eLife.74441 (PMC8752092; doi:10.7554/eLife.74441)
Supplement: Transparent reporting form [file elife-74441-transrepform1.docx]

***eLife’s* transparent reporting form**

We encourage authors to provide detailed information *within their submission* to facilitate the interpretation and replication of experiments. Authors can upload supporting documentation to indicate the use of appropriate reporting guidelines for health-related research (see [EQUATOR Network](http://www.equator-network.org/%20)), life science research (see the [BioSharing Information Resource](https://biosharing.org/)), or the [ARRIVE guidelines](http://www.plosbiology.org/article/info:doi/10.1371/journal.pbio.1000412) for reporting work involving animal research. Where applicable, authors should refer to any relevant reporting standards documents in this form.

If you have any questions, please consult our Journal Policies and/or contact us: [editorial@elifesciences.org](mailto:editorial@elifesciences.org).

**Sample-size estimation**

- You should state whether an appropriate sample size was computed when the study was being designed
- You should state the statistical method of sample size computation and any required assumptions
- If no explicit power analysis was used, you should describe how you decided what sample (replicate) size (number) to use

Please outline where this information can be found within the submission (e.g., sections or figure legends), or explain why this information doesn’t apply to your submission:

For confocal images 4-6 images from 4 different tissue sections per mouse, with 2 mice per genotype were utilised in order to cover the biological variability between individual synapses throughout the spinal cord tissue. For super-resolution images 6-14 images from 3 different tissue sections for both dorsal and ventral regions, with 2 mice per age per genotype giving a total of 10-23 images and 120-433 synapses per condition were utilised to cover the biological variability between individual synapses. A total of 38 synapses from 5 different tissue sections from the 10 month super-resolution dataset were utilised for electron microscopy. All sample sizes are stated in the figure legends.

**Replicates**

- You should report how often each experiment was performed
- You should include a definition of biological versus technical replication
- The data obtained should be provided and sufficient information should be provided to indicate the number of independent biological and/or technical replicates
- If you encountered any outliers, you should describe how these were handled
- Criteria for exclusion/inclusion of data should be clearly stated
- High-throughput sequence data should be uploaded before submission, with a private link for reviewers provided (these are available from both GEO and ArrayExpress)

Please outline where this information can be found within the submission (e.g., sections or figure legends), or explain why this information doesn’t apply to your submission:

All replicates are stated in all figure legends, including both technical and independent biological replicates. No data were excluded from analyses. Reproducibility was verified and confirmed by comparing confocal and super-resolution synapse data between WT animals and between different ages, and with WT littermates of oscillator mice. Further, mice were compared for reproducibility when calculating the molecule conversion from PALM images. All these values are reported in the study.

**Statistical reporting**

- Statistical analysis methods should be described and justified
- Raw data should be presented in figures whenever informative to do so (typically when N per group is less than 10)
- For each experiment, you should identify the statistical tests used, exact values of N, definitions of center, methods of multiple test correction, and dispersion and precision measures (e.g., mean, median, SD, SEM, confidence intervals; and, for the major substantive results, a measure of effect size (e.g., Pearson's r, Cohen's d)
- Report exact p-values wherever possible alongside the summary statistics and 95% confidence intervals. These should be reported for all key questions and not only when the p-value is less than 0.05.

Please outline where this information can be found within the submission (e.g., sections or figure legends), or explain why this information doesn’t apply to your submission:

All statistical analysis methods are described in the relevant figure legends, and are further described and justified in the ‘Graphing and statistical analysis’ section of the Materials and Methods. Raw data is presented in the figures, where the use of dot plots show all raw values. For each experiment the values of N, the statistical tests, methods of multiple test correction, precision measures (mean, median, SD, SEM) and measure of effect size (Spearman rank) are identified in each figure legend, as well as median values and Spearman rank r in text within the figures. P-values are indicated in the figures and described in the figure legends where necessary.

(For large datasets, or papers with a very large number of statistical tests, you may upload a single table file with tests, Ns, etc., with reference to sections in the manuscript.)

**Group allocation**

- Indicate how samples were allocated into experimental groups (in the case of clinical studies, please specify allocation to treatment method); if randomization was used, please also state if restricted randomization was applied
- Indicate if masking was used during group allocation, data collection and/or data analysis

Please outline where this information can be found within the submission (e.g., sections or figure legends), or explain why this information doesn’t apply to your submission:

Mice were selected based on their genotype to ensure all analysis and controls could be carried out. All super-resolution images were batch analysed, including images taken on different dates and from different regions of the spinal cord, thus the investigator was blind to raw data until after analysis. All electron microscopy data was imaged and analysed blind.

**Additional data files (“source data”)**

- We encourage you to upload relevant additional data files, such as numerical data that are represented as a graph in a figure, or as a summary table
- Where provided, these should be in the most useful format, and they can be uploaded as “Source data” files linked to a main figure or table
- Include model definition files including the full list of parameters used
- Include code used for data analysis (e.g., R, MatLab)
- Avoid stating that data files are “available upon request”

Please indicate the figures or tables for which source data files have been provided:

Source data are provided for the full size gels in Figure 1, Supplementary file 1.
